# Bacteriophage Isolated from Sewage Eliminates and Prevents the Establishment of *Escherichia Coli* Biofilm

**DOI:** 10.15171/apb.2018.011

**Published:** 2018-03-18

**Authors:** Karla Veloso Gonçalves Ribeiro, Cleberson Ribeiro, Roberto Sousa Dias, Silvia Almeida Cardoso, Sergio Oliveira de Paula, Jose Cola Zanuncio, Leandro Licursi de Oliveira

**Affiliations:** ^1^Núcleo de Microscopia e Microanálise, Universidade Federal de Viçosa, 36570-900, Viçosa, Minas Gerais, Brasil.; ^2^Departamento de Biologia Geral, Universidade Federal de Viçosa, 36570-900, Viçosa, Minas Gerais, Brasil.; ^3^Departamento de Medicina e Enfermagem, Universidade Federal de Viçosa, 36570-900, Viçosa, Minas Gerais, Brasil.; ^4^Departamento de Entomologia, Universidade Federal de Viçosa, 36570-900, Viçosa, Minas Gerais, Brasil.

**Keywords:** Biocontrol, Biofilm, E. coli, Phage

## Abstract

***Purpose:*** Biofilm growth exerts a negative impact on industry and health, necessitating the development of strategies to control. The objective of this work was study the lytic activity of the phage isolated from the sewage network in the formation and degradation of Escherichia coli biofilms.

***Methods:*** E. coli cultures were incubated in 96-well polystyrene microplates under controlled conditions to evaluate the biofilm formation. The E. coli cultures and established biofilms were treated with the suspensions of the vB_EcoM-UFV017 (EcoM017) bacteriophage obtained from sewage for 24 hours. The E. coli bacterial density was measured using absorbance at 600 nm and the biofilms were measured by crystal violet staining. Polystyrene coupons were used as support for Scanning Electron Microscopy and Confocal Microscopy to evaluate biofilm formation.

***Results:*** The E. coli strains formed biofilms in polystyrene microplates after 48 hours’ incubation. The highest EcoM017 phage titer, in the prevention and degradation experiments, reduced the bacterial growth and the quantity of biofilm formed by E. coli in 90.0% and 87.5%, respectively. The minimum dose capable of reducing the biofilms of this bacterium was 10^1^ PFU/mL after 24 hours. The preformed E. coli biofilm mass was reduced 79% post exposure to the phage in the degradation assay. Microscopic analysis confirmed the results obtained in the plates assays.

***Conclusion:*** The EcoM017 phage prevented biofilm formation and degraded the E. coli-established ones. The EcoM017 phage isolated from sewage can reduce bacterial attachment and lyse the E. coli associated biofilm cells, offering biotechnological potential applicability for this phage.

## Introduction

*Escherichia coli,* one of the main components of the normal flora of the digestive tract of humans and animals,^1^ are usually harmless, although some strains are pathogenic and can cause intra- and extra-intestinal diseases.^2^ Specific gene groups and virulence factors facilitate the colonization, multiplication and survival of this bacterium in the host body.^3^

*E. coli* can adhere to and be internalized in epithelial cells. The persistence of this pathogen in cattle mammary glands causes an infection called mastitis, whose recurrence may be related to biofilm formation.^4^ Bovine mastitis is the most significant and expensive disease affecting the dairy industry across the world.^5^ *E. coli* is the most important and frequently agent isolated from the clinical cases of environmental mastitis causing milk production losses and the death of 10% of the animals.^6,7^ This organism is capable of causing clinical mastitis depending on the immunological status of animal.^8^

Biofilms are complex microbial communities found adherent to a surface and embedded in a protective extracellular matrix (ECM) of polymeric substances.^9^ Biofilm formation increases resistance to antibiotics, disinfectants, phages and the host immune system, including antibodies, phagocytes and the complement system, necessitating the development of strategies to control their formation.^10^ Since the 20th, phages have been proposed as antimicrobial agents.^11^ However, the discovery and commercialization of antibiotics from the ’40s, emergence of broad-spectrum antibiotics, lack of quality therapeutic products and less understanding of the biology delayed or resulted in minimum research done on phages.^12^

Inappropriate antibiotic use increased the emergence of resistant bacteria and rekindled the interest in phage therapy.^13,14^ Phages are highly specific to one or a few bacteria and are often effective against multi-drug resistant organisms. This is because they kill cells via different molecular mechanisms without infecting eukaryotic cells or disrupting the normal host beneficial microflora.^15,16^ Therefore, these organisms have been proposed to remove or reduce bacterial biofilm.^17^

The EcoM017 phage isolated from sewage has been tested for *E. coli* biocontrol isolated from animals with mastitis, aiming at preventing biofilm formation and degrading those already established by these bacteria.

## Materials and Methods

### Bacterial strain

*Escherichia coli* 30 were isolated from cow with mastitis. This strain is part of the collection of pathogens from the Laboratory of Molecular Immunovirology (LMI) of the Departamento de Biologia Geral, Universidade Federal de Viçosa (UFV) and was obtained from Embrapa Dairy Cattle. The bacteria were grown in Luria-Bertani agar (LB) (10 g/L tryptone, 5 g/L yeast extract, 10 g/L NaCl (pH 7.2) and supplemented with 1.5% bacteriological agar) at 37°C for 16 h. One colony was isolated and transferred to the LB broth (without agar supplementation) and cultivated at 37°C under stirring (180 rpm) until an optical density of 0.7 at a wavelength of 600 nm (OD_600_) was achieved in the microplate reader (VersaMax, Molecular Device). The bacterial culture was centrifuged (4,000 × g) for 10 min. The pellet was suspended and adjusted to OD_600_ 2.0 in 10 mM MgSO_4_ . The stocks were refrigerated at 4°C and used for titration and plating of the phage.

### Phage propagation

The EcoM017 phage was isolated from the sewage network samples in Viçosa municipality, Minas Gerais state, Brazil, and used in the experiments for the prevention and degradation of the *E. coli* 30 biofilm. Phage stocks were prepared using the standard agar-overlay technique. A total of 100 µL of the bacterial stock with 2.0 OD_600_ was added to 100 µL of the viral stock. This mixture was incubated at 37°C for 30 min and added to tubes containing semi-solid agar supplemented with 0.75% bacteriological agar. The entire tube contents were transferred onto a plate with LB agar. The phages were extracted in 5 mL SM buffer (100 mM NaCl, 8 mM MgSO_4_.7H_2_O, 50 mM Tris-HCl pH 7.5, 2% gelatin w/v) at 4°C with gentle agitation for 4 h, after 24 h incubation. The SM buffer was recovered, supplemented with 1 M NaCl and maintained at 4°C for 1 h. The contaminant bacteria were removed by passage through a sterile 0.22 µm filter. The EcoM017 phage stocks were stored at 4°C for use.

### Phage characterization and titration

The morphology of the phage was observed by Transmission Electron microscopy (TEM). Briefly, the purified phage sample was deposited on Formvar-coated copper grid and negative stained with 2% uranyl acetate. Visualization was performed using a Zeiss TEM EM 109 (Zeiss, Oberkochen, Germany) at an accelerating voltage of 80 kV. The phage titer was analyzed with the standard agar-overlay technique through the serial dilution of stocks and calculation of plaque-forming units (PFU) per mL (PFU/mL). First, 100 µL of the phage diluted solution was added to 100 µL of the bacterial stock with 2.0 OD_600_ and incubated in plates with LB agar as described above (Phage propagation). Plate that has between 30 and 300 lysis plaques were used to calculate Plaque Forming Units per mL, using the equation:

PFU mL^-1^ = number of lysis plaques X dilution factor / volume of diluted virus added to the plate

### Quantification of biofilm formation

The detection and quantification of the *E. coli* biofilm were performed in 96-well polystyrene microplates. This bacterium was cultivated under shaking at 180 rpm at 37°C for 2 h. The OD_600_ of the *E. coli* culture was adjusted to 0.1, corresponding to approximately 10^8^ colony forming units (CFU) per mL,^18,19^ and the culture was diluted 1:10 in fresh LB broth and inoculated into six wells per microplate (200 µL per well). The control had wells containing sterile LB broth. The plates were incubated at 37°C for 24, 48 and 72 h respectively, to allow biofilm development to occur. Post incubation, bacterial growth (OD_600_) was determined in the microplate reader. Planktonic cells were removed by quickly inverting the plate and each well was rinsed twice with 250 µL of phosphate buffered saline (PBS) (pH 7.2). The adherent cells were stained with 250 µL of 0.1% crystal violet (CV) at room temperature for 30 min. The wells were washed four times with 250 µL distilled water and air dried for 1 h. The CV was solubilized in 200 µL of acetone-ethanol 20:80 at room temperature. The amount of CV extracted was measured by optical density at 595 nm (OD_595_) in the microplate reader.

### Biofilm prevention with phage

A total of 20 µL of bacterial culture with 0.1 OD_600_ and 20 µL phage suspension at different concentrations (concentrated solution and nine decimal dilutions in SM buffer) were added to 160 µL of the LB broth in the wells of the 96-well polystyrene microplates. The control contained the LB broth, LB plus phage and LB plus bacterial suspension. The plate was incubated at 37°C for 24 h. Post incubation, the OD_600_ was determined and the biofilm was stained with CV, as described above. The relative biofilm mass was determined by normalizing the absorbance of the positive control at 100%.

### Biofilm degradation with phage

Initially 20 µL of the bacterial culture with 0.1 OD_600_ of was added to 180 µL of the LB broth in the wells of a 96-well polystyrene microplate. The control contained LB and LB plus bacterial suspension. The microplates were incubated at 37°C for 24 h. The wells were washed twice with PBS and 20 µL of the phage solution at different concentrations (concentrated solution and nine decimal dilutions in SM buffer) and 180 µL of the LB broth were added. The controls contained the LB broth, LB plus phage and LB plus bacterial suspension. The microplates were incubated at 37°C for 24 h. The OD_600_ was determined in the microplate reader and the CV stained wells as described above. The relative biofilm mass was determined by normalizing the absorbance of the positive control at 100%.

### Preparation of polystyrene coupons

Polystyrene pieces (coupons) (1.0 × 1.0 × 0.1 cm) were used as the support for the biofilm formation. Each coupon was brushed with detergent, rinsed in distilled water, sterilized by immersion in 1% sodium hypochlorite and 70% ethanol for 15 min, and placed under ultraviolet light for 30 min, on each side.

### Biofilm formation

The coupons were placed in 15 mL Falcon tubes containing 125 μL of the bacterial culture with 0.1 OD_600_ and 1,125 μL of the LB broth (coupons at the air-liquid interface). The control coupons were immersed in LB alone. The tubes were incubated at 37°C without shaking for 24, 48 and 72 h, and prepared for analysis by Scanning Electron Microscopy (SEM).

### Phage effect on biofilm formation

The coupons were placed in 15 mL Falcon tubes containing 125 µL of the bacterial culture with 0.1 OD_600_, 1 mL LB broth and 125 µL of the phage solution with three titers: high (10^10^ PFU/mL), medium (10^4^ PFU/mL) and low (10^1^PFU/mL). The control coupons were immersed in the LB broth, LB plus SM buffer and LB plus bacterial suspension. The tubes were incubated at 37°C without stirring for 24 h. The coupons were prepared for analysis by the SEM (coupons treated with three phage titers) and Confocal Laser Scanning Microscopy (CLSM) (coupons treated with high and low titers) as described below.

### Phage effect on the biofilm

The coupons were placed in 15 mL Falcon tubes with 125 µL of bacterial culture with 0.1 OD_600_ and 1,125 µL LB broth. The control coupons were immersed in the LB, LB plus SM buffer and LB plus bacterial suspension. The tubes were incubated at 37°C without stirring for 24 h. After establishment of the biofilm, the coupons were washed twice with PBS (pH 7.2) and transferred to the tubes containing 125 µL of phage solution with high (10^10^ PFU/mL), medium (10^4^ PFU/mL) and low (10^1^ PFU/mL) and 1,125 µL of LB. The tubes were incubated at 37°C without agitation for 24 h and the coupons prepared for analysis by SEM (coupons treated with three phage titers) and CLSM (coupons treated with high and low titers).

### Scanning Electron Microscopy (SEM)

The coupons were washed in PBS (pH 7.2), fixed with 2.5% glutaraldehyde v/v in 0.05 M cacodylate buffer (pH 7.2) for 2 h and treated with 1% (w/v) osmium tetroxide solution in 0.05 M cacodylate buffer (pH 7.2) at room temperature for 1 h. After cell fixation, the samples were rinsed thrice in 0.05 M cacodylate buffer and immersed in an increasing ethanol gradient (30, 50, 70, 80 and 90% in water) for 10 min per concentration. Finally, the samples were immersed thrice in 100% ethanol for 10 min, and then dehydrated, dried in a dryer critical point (CPD Bal-tec 030) with liquid CO_2_ and covered with gold (15 nm thick approximately) in a sputter (Balzers, FDU 010). The coupons were examined in a Scanning Electron Microscope (Leo 1430VP) with an accelerating voltage of 20 kV.

### Confocal Laser Scanning Microscopy (CLSM)

The coupons were washed twice in PBS (pH 7.2) and incubated in a mixture of propidium iodide (IP) 20 µg/mL and fluorescein isothiocyanate 2 µg/mL in PBS (pH 7.2), for 15 min in the dark.^20^ Post staining, the coupons were washed in PBS and examined under the Laser Scanning Confocal Microscope (Zeiss LSM 510 Meta) with argon laser excitation at 488 and 514 nm. The live bacteria with intact membranes would stain green, whereas dead and membrane-damaged ones would stain red or yellow.

### Statistical analysis

The results of three independent experiments performed in sextuplicate were subjected to variance analysis (ANOVA) and Tukey test using the GraphPad Prism Software, Inc. Statistical significance was accepted if P ≤ 0.05.

## Results

### Phage Characterization

**Figure 1:**
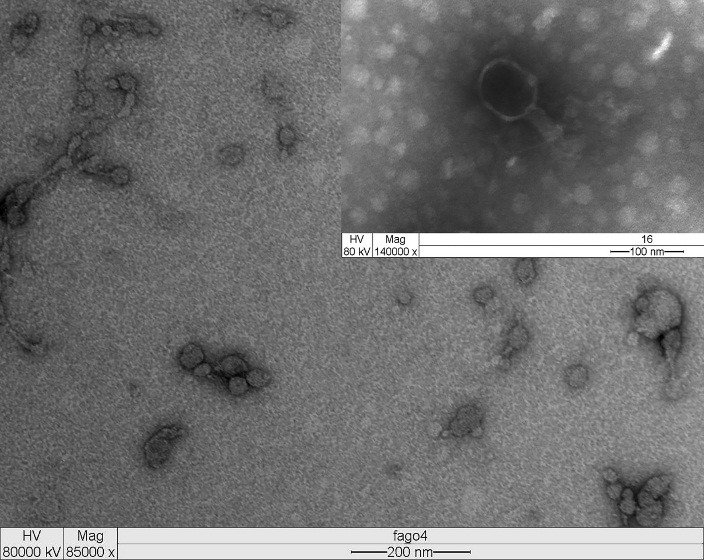
Transmission Electron micrograph of bacteriophage vB_EcoM-UFV017. The phage showing an isometric head structure with ~ 89 nm in length and ~78 nm in width and a tail of ~55 nm. The morphology of vB_EcoM-UFV017 corresponds to the Myoviridae family. The insert show details of phage morphology (head, tail, base plate and tail fibers). Virion particles were negatively stained with uranyl acetate for EM.

The phage isolated from the sewage was processed for Transmission electron microscopy (TEM), the phage revealed an isometric head (~ 89 nm in length and ~78 nm in width) and contractile tails (~ 55 nm) with a basal tuft attached (Figure1). Based on these morphological characteristics, phage was assigned to the family Myoviridae according to the classification system of Ackermann^21^ and named vB_EcoM-UFV017. Myoviridae phages typically possess double-stranded (ds) DNA as their genomic nucleic acid. In addition, the genome size of phage was approximately 44 kb (unpublished data).

### Biofilm formation

**Figure 2:**
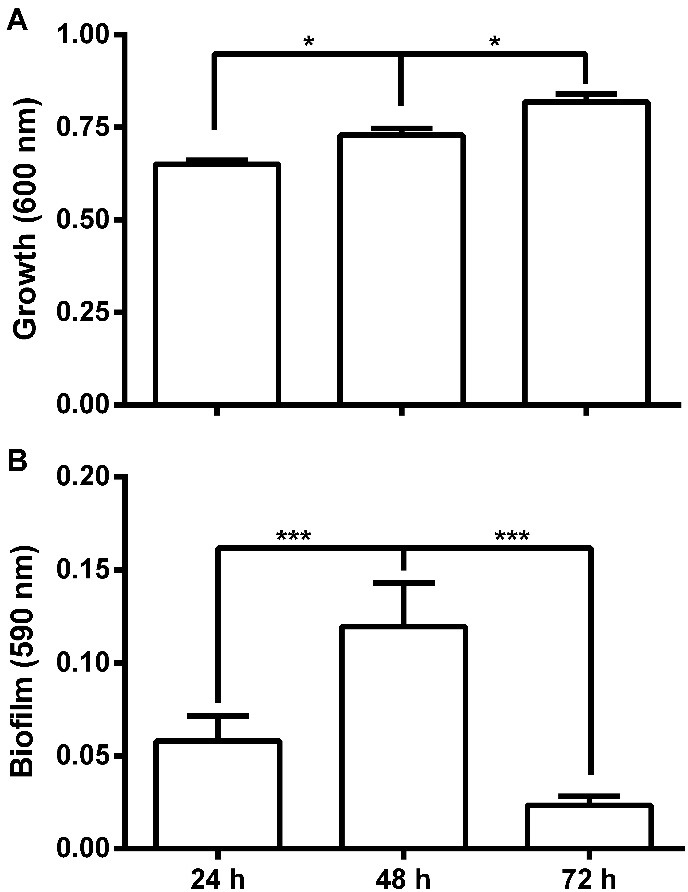
Bacterial growth (A) and biofilm quantification (B) of *Escherichia coli* 30 in polystyrene microplates after incubation in LB at 37 °C under static conditions for 24, 48 and 72 hours. * *P* <0.05 and *** P <0.001.

**Figure 3:**
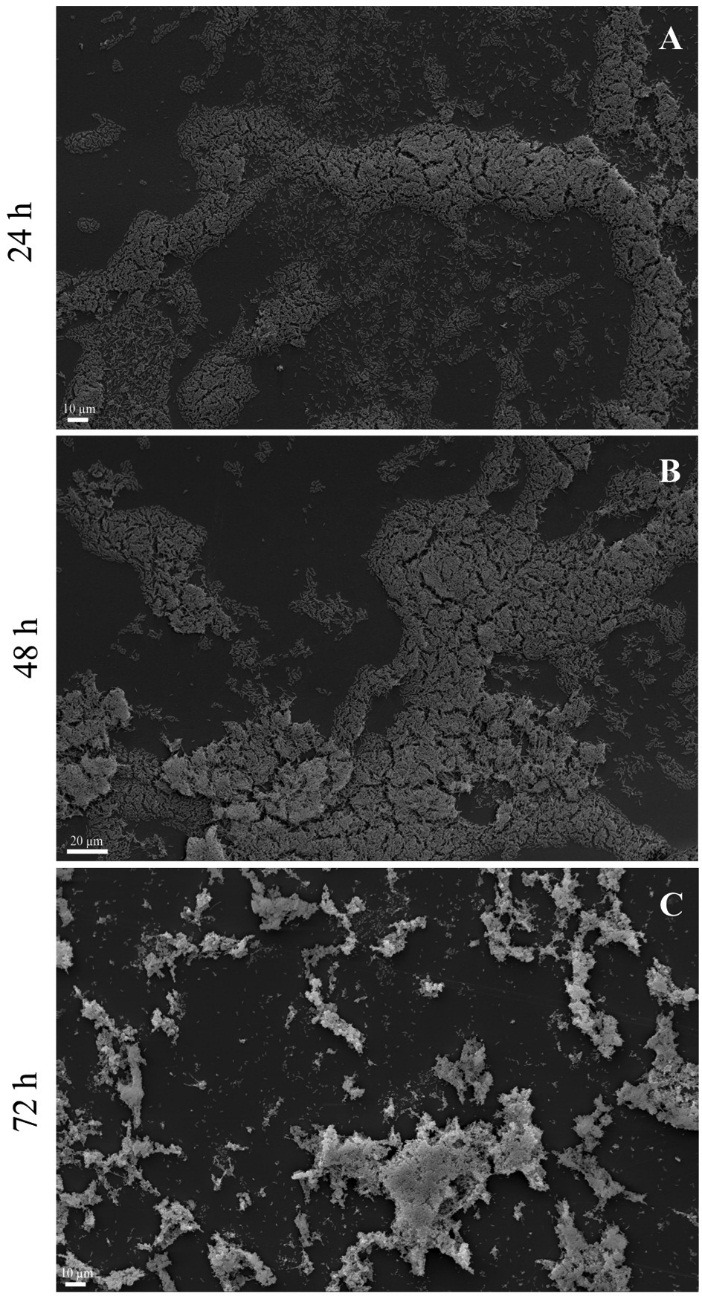
Scanning electron microscopy of *Escherichia coli* 30 biofilm grown on the polystyrene surface under static conditions at 37 °C for 24, 48 and 72 hours.

*Escherichia coli* formed biofilm on the polystyrene microplate in the LB medium at 37°C under static conditions for 24 and 48 hours, with a two-fold increase between these periods. At 72 hours, the biofilm mass was 5.4 times lower than that of the one at 48 hours (Figure 2) possibly due to nutrient depletion and other factors (see Discussion). The scanning electron microscopy showed similar results to those of colorimetric quantitative assays (Figure 3).

### Effect of the phage on biofilm formation

**Figure 4:**
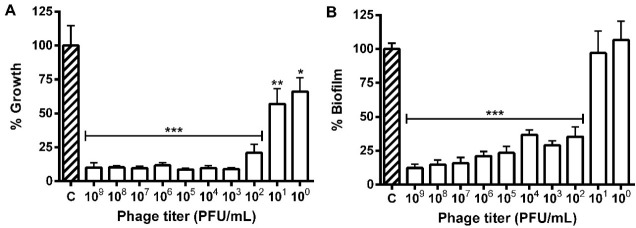
Prevention of the growth (A) and biofilm formation (B) of *Escherichia coli* 30 in polystyrene microplates after 24 hours of exposure to different phage titers (10^0^ to 10^9^ PFU/mL). * P ≤ 0.05, ** P ≤ 0.01 and *** P ≤ 0.001 compared to the control.

The different phage titers decreased the *E. coli* population and those with 10^2^ to 10^9^ PFU/mL reduced the bacterial growth and biofilm formation. The highest titer (10^9^ PFU/mL) reduced the growth and biofilm formation in 90.0 and 87.5%, respectively, while the 10^0^ and 10^1^ PFU/mL biofilms showed no difference from control (Figure 4).

**Figure 5:**
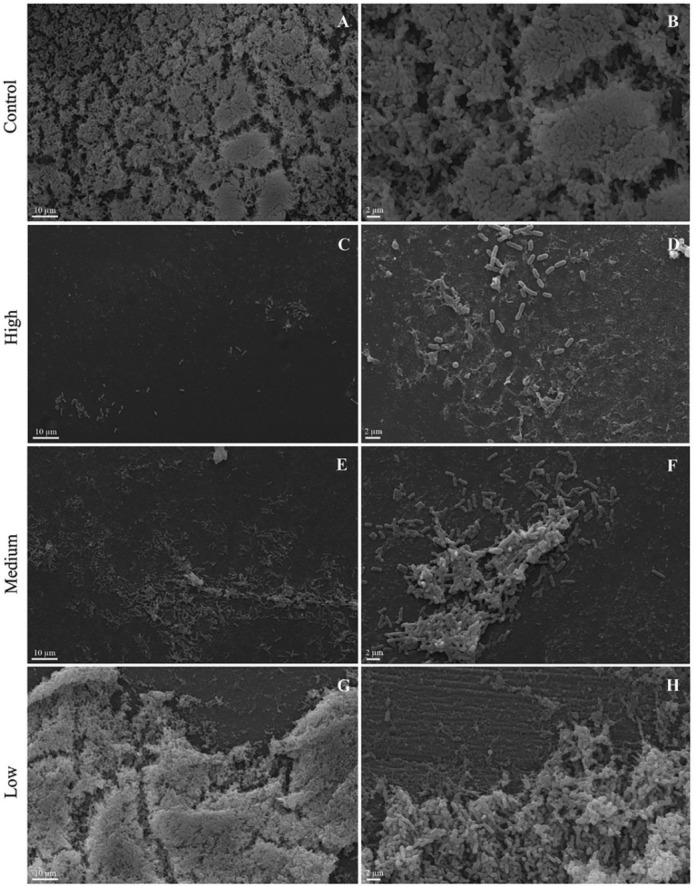
Scanning electron microscopy of *Escherichia coli* 30 biofilm grown on the polystyrene surface under static conditions at 37 °C for 24 hours. Control untreated (A and B). Treatment with phage with a high titer (10^9^ PFU/mL) (C and D), medium titer (10^4^ PFU/mL) (E and F) and low titer (10^0^ PFU/mL) (G and H).

**Figure 6:**
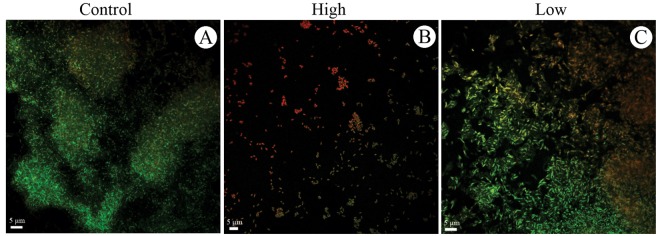
Confocal microscopy of *Escherichia coli* 30 biofilm grown on the polystyrene surface under static conditions at 37 °C for 24 hours. Prevention experiment: (A) Control untreated, (B) Treatment with phage solution with a high titer (10^9 PFU/mL) and (C) low titer (10^0 PFU/mL).

*Escherichia coli* formed biofilm on the polystyrene coupon with large cell groups, a small amount of ECM and the prevalence of viable cells (green color) when incubated at 37°C under static conditions for 24 hours (Figures 5A, 5B and 6A).

Medium and high titer treatments decreased the biofilm formation of *E. coli*, showing a dose-dependent effect. Single cells and small clusters containing large numbers of dead cells (red staining) were observed in the treatment with the highest titer. Cell debris was also observed in both treatments (Figures 5C-5F and 6B).

Debris and a greater number of dead cells were observed near the biofilm in the presence of low titer of the phage (10^1^ PFU/mL) (Figures 5G, 5H and 6C).

### Effect of the phage on biofilm degradation

**Figure 7:**
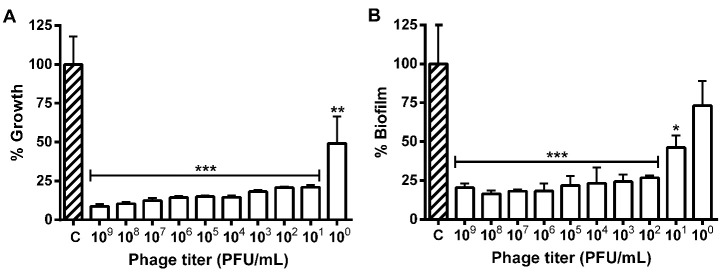
Growth of cell attached (A) and degradation of preformed biofilm (24h) (B) of *Escherichia coli* 30 in polystyrene microplates after 24 hours of exposure to different phage titres (10^0^ to 10^9^ PFU/mL). * P ≤ 0.05, ** P ≤ 0.01 and *** P ≤ 0.001 compared to the control.

The minimum dose capable of reducing the *E. coli* 30 biofilm was found to be 10^1^PFU/mL. The biofilm formation dropped to 79.0% in the treatment with the highest phage titer (10^9^PFU/mL) (Figure 7)

**Figure 8:**
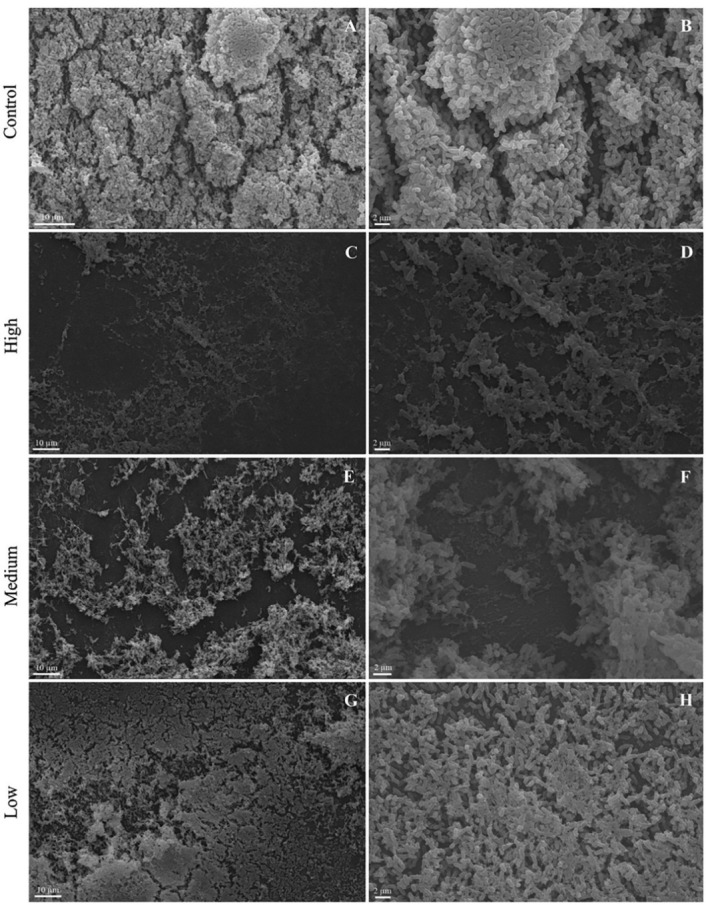
Scanning electron microscopy of *Escherichia coli* 30 biofilm grown on the polystyrene surface under static conditions at 37 °C for 24 hours. Control untreated (A and B). Treatment with phage with a high titer (10^9^ PFU/mL) (C and D), medium titer (10^4^ PFU/mL) (E and F) and low titer (10^0^ PFU/mL) (G and H).

**Figure 9:**
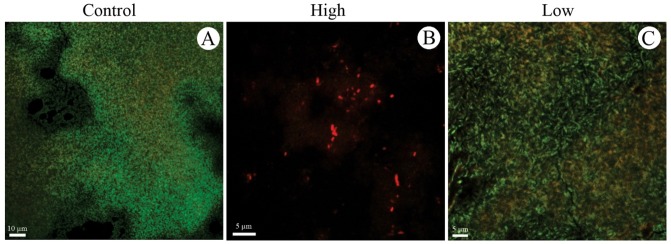
Confocal microscopy of *Escherichia coli* 30 biofilm grown on the polystyrene surface under static conditions at 37 °C for 24 hours. Degradation experiment: (A) Control untreated, (B) Treatment with phage solution with a high titer (10^9 PFU/mL), and (C) low titer (10^0 PFU/mL).

*E. coli* grown in the absence of the phage yielded a complex biofilm with a predominance of viable cells (Figures 8A, 8B and 9A). The medium and high phage titers destroyed the architecture of the *E. coli* biofilm in a dose-dependent manner, revealing a predominance of dead cells (Figures 8C-8F and 9B). The low titer of the phage (10^1^ PFU/mL) exerted no effect on the mature biofilm of* E. coli* (Figures 8G, 8H and 9C).

## Discussion

The increasing inefficiency of antibiotic therapy to eliminate biofilm-associated infections has accelerated the search for alternative therapeutic agents, including phages.^22^ Moreover, it has been shown that *E. coli* 30 can form more biofilm when grown in media with enrofloxacin, which is commonly used in the treatment of *E. coli* bovine mastitis, showing the importance to develop alternatives to the treatment of mastitis caused by this isolate.^23^

There is remarkable variation in biofilm formation by *E. coli* isolates *in vitro*.^24^ The biofilm formation by *E. coli* 30 on the polystyrene after 24 hours’ incubation is related to the ability of these bacteria to adhere to biotic and abiotic surfaces by physicochemical, molecular and cellular interactions. This action is mediated by the bacterial cell wall appendages, such as fimbriae type 1 and conjugative pili under different temperatures, pH, aerations and nutrient conditions.^25^ The high production of biofilm by the *E. coli* 30 strain may be related to nutrient-rich-medium (LB) that ensure the necessary nutrients for cell proliferation and matrix production whereas in nutritionally-limited R2A medium, *E. coli* formed small clusters composed of isolated cells.^26^

Bacteria grown with renewal of media forms a big amount of biomass mature biofilm in microtiter plate.^27^ The reduction in the *E. coli* 30 biofilm mass in the 72-hour period may be associated with cell death caused by a drop in the nutrient availability, lack of aeration and metabolite accumulation, resulting in the detachment of the biofilm from the substrate due no media renewal.^28,29^ The stress precipitated by hypoxia and nutrient limitation promotes the expression of those genes involved in biofilm dispersion, essential for the bacteria to escape the unfavorable conditions and colonize a new environment.^30,31^

Qualitative analysis of scanning electron microscopy results confirmed the results obtained for quantitative analyses and showed that *E. coli* 30 formed structured biofilm for 48h and *unstructured* biofilm composed of isolated cell clusters after 72h. This shows that SEM is a good quality control of biofilm formation on different surfaces.^32^

Some studies have demonstrated the significant potential of phages to reduce and/or eliminate biofilms.^33^ This was reported for a phage mixture where *Escherichia coli* O157:H7 biofilms formed on different surfaces, times and temperatures showed pronounced decrease in its biofilm after addition of phage mixture.^34^ In our experiments the EcoM017 phage was able reduce significantly the cell growth and biofilm formed on polystyrene surface. The reduction is due to phage capacity of adsorbing on the bacterial cell surface, inject its genetic material and use the metabolic machinery of the host to multiply, resulting in cell lysis and releasing new particles inside biofilm.^35^ The greatest reduction in the bacterial growth with respect to the biofilm was observed in the highest titer of the phage (10^9^ PFU/mL). This is related to the higher susceptibility of the planktonic cells (free) to the phage that are metabolically more active than biofilm cells.^36^

The low quantity of ECM observed can be linked to the *E. coli* growth under inadequate conditions of oxygen (no agitation) and matrix removal during coupon preparation for SEM and can facilitate phage penetration into biofilm.^37^ It has been shown that cells at the air-biofilm interface are more evenly spaced and often not surrounded by a fibrous extracellular matrix.^38^ The predominance of the viable cells in the hydrated biofilm analyzed by Confocal Microscopy shows a natural balance between the living and dead cells within the biofilm, in which the first stained green (FITC) and the second red (IP penetrated through the damaged membranes).

The presence of single cells and small cell groups along with the dead ones, besides the cellular debris in the biofilms formed when the high titer of the EcoM017 phage was used, is due to phage multiplication-induced pronounced cell lysis. Phage multiplication in biofilms can limit spread of biofilm and maintain cells at density low enough to clear the infection by immune response; however, *E. coli* biofilms treated over several days can develop phage resistance.^39^ Alternatively, suspensions that are more concentrated could be used to eliminate the biofilm in a shorter time. Similar efficiency in preventing colonization of the surfaces was observed for *Escherichia coli* K12 and *Pseudomonas aeruginosa* with specific lytic phages.^40,41^

Cellular debris found in the biofilm edges during the low phage titer treatment is due the beginning of cell lysis at the ends of the biofilm as reported for *Pseudomonas fluorescens* grown in stainless steel coupons in the presence of the phage-phiIBB PF7A.^42^

The reduced biofilm formation can be related to the capacity of the EcoM017 phage to access the biofilm and infect the bacteria within causing cell death and degrading the established biofilms.^43^ The phage isolated from the sewage also curtailed the biofilm formation by *E. coli* O157: H7 in steel coupons, although the phage concentration used was 10^6^ times higher.^44^

The high degradation of the biofilm (79%) with the highest phage titer can be explained by the affinity of this last treatment for the bacterium and the low quantity of ECM present; it has also been reported that the use of the lytic phage in the catheter prevented *E. coli* biofilm formation associated with urinary tract infections.^45^ Similar results were observed in *Klebsiella* sp. biofilms treated with the phage isolated from sewage.^46^

Our findings reveal that EcoM017 phage can destroy biofilm architecture due to the aqueous channels found between the cell groups facilitating access to the surface of the bacteria, penetrating even to the deeper layers. So EcoM017 phage to lyse the bacteria at different layers and detach parts of the biofilm in the liquid medium. The detached cells after being released from the groups to the planktonic phase are more susceptible to phage attack.^47^ Dead cells and cell debris were also observed in the *Pseudomonas aeruginosa* biofilm treated with the phage isolated from sewage; however, the biofilm architecture revealed fewer alterations.^48^

Phages can be applied to inhibit the adhesion of bacteria, lysing them and favoring the action of antibiotics. EcoM017 phage suspension may be applied in inoculation via the teat canal similarly to treatment with antibiotics to reduce the bacterial attachment and lyse the cells associated with *E. coli* 30 biofilm and accordingly can be used to control of bovine mastitis.

## Acknowledgments

The authors would like to thanks the ‘Fundação de Amparo à Pesquisa do Estado de Minas Gerais - FAPEMIG’ and ‘Núcleo de Microscopia e Microanálise - NMM’ of the Universidade Federal de Viçosa.

## Ethical Issues

Not applicable.

## Conflict of Interest

Authors declare no conflict of interest in this study
